# Sensitivity of Hyperdense Basilar Artery Sign on Non-Enhanced Computed Tomography

**DOI:** 10.1371/journal.pone.0141096

**Published:** 2015-10-19

**Authors:** Marielle Ernst, Javier M. Romero, Jan-Hendrik Buhk, Bastian Cheng, Jochen Herrmann, Jens Fiehler, Michael Groth

**Affiliations:** 1 Center for Radiology and Endoscopy, Department of Diagnostic and Interventional Neuroradiology, University Medical Center Hamburg-Eppendorf, Hamburg, Germany; 2 Department of Radiology, Division of Neuroradiology, Massachusetts General Hospital, Harvard Medical School, Boston, Massachusetts, United States of America; 3 Department of Neurology, University Medical Center Hamburg-Eppendorf, Hamburg, Germany; 4 Center for Radiology and Endoscopy, Department of Diagnostic and Interventional Radiology, Division of Pediatric Radiology University Medical Center Hamburg-Eppendorf, Hamburg, Germany; University Medical Center (UMC) Utrecht, NETHERLANDS

## Abstract

**Purpose:**

The hyperdense basilar artery sign (HBAS) is an indicator of vessel occlusion on non contrast-enhanced computer tomography (NECT) in acute stroke patients. Since basilar artery occlusion (BAO) is associated with a high mortality and morbidity, its early detection is of great clinical value. We sought to analyze the influence of density measurement as well as a normalized ratio of Hounsfield unit/hematocrit (HU/Hct) ratio on the detection of BAO on NECT in patients with suspected BAO.

**Materials and Methods:**

102 patients with clinically suspected BAO were examined with NECT followed immediately by Multidetector computed tomography Angiography. Two observers independently analyzed the images regarding the presence or absence of HBAS on NECT and performed HU measurements in the basilar artery. Receiver operating characteristic curve analysis was performed to determine the optimal density threshold for BAO using attenuation measurements or HU/Hct ratio.

**Results:**

Sensitivity of visual detection of the HBAS on NECT was relatively low 81% (95%-CI, 54–95%) while specificity was high 91% (95%-CI, 82–96%). The highest sensitivity was achieved by the combination of visual assessment and additional quantitative attenuation measurements applying a cut-off value of 46.5 HU with 94% sensitivity and 81% specificity for BAO. A HU/Hct ratio >1.32 revealed sensitivity of 88% (95%-CI, 60–98%) and specificity of 84% (95%-CI, 74–90%).

**Conclusion:**

In patients with clinically suspected acute BAO the combination of visual assessment and additional attenuation measurement with a cut-off value of 46.5 HU is a reliable approach with high sensitivity in the detection of BAO on NECT.

## Introduction

The hyperdense basilar artery sign (HBAS) was first described in 1982 by Kuckein et al. as hyperattenuation of the basilar artery (BA) on non-contrast enhanced computed tomography (NECT) scans representing basilar artery occlusion (BAO).[1] Early diagnosis is vital since BAO is associated with a high mortality and morbidity rate between 80 and 90% without timely detection and recanalization.[2,3]

The unspecific, and partly fluctuating symptoms in up to >90% of the patients often cause delays to the correct diagnosis. Many patients with BAO are primarily admitted to community medical centers that do not routinely perform advanced imaging such as Multidetector Computed Tomography Angiography (MDCTA), Magnetic Resonance Angiography (MRA) or Digital Subtraction Angiography (DSA). Thus determining the most sensitive approach of defining HBAS on NECT for the diagnosis of BAO is of great clinical value in the guiding of further treatment or confirmatory test. Moreover determining the diagnostic accuracy of different imaging methods is essential in the field of health economics as sensitivity and specificity are used as input parameters for cost effectiveness analysis in the diagnostic work-up of possible BAO.[4]

We therefore aimed to investigate the influence of density measurement on the detection of BAO on NECT in patients with clinically suspected BAO. In addition to previous studies [5–7], we analyzed if a normalized ratio of HU/Hct (correction for Hct) further increases sensitivity as x-ray attenuation is highly correlated with hematocrit levels and an increased hematocrit (Hct) might mimic the HBAS.

We hypothesized that measuring intra-arterial thrombus density improves the sensitivity for the diagnosis of BAO compared with visual assessment alone.

## Materials and Methods

### Patient selection

We identified 123 consecutive patients presenting to our department with suspect of BAO between January 2010 and March 2012. In our institution BAO is considered, if one of the following clinical signs was present:

sudden onset of

- nontraumatic impaired consciousness
- Motor deficits such as hemiparesis or tetraparesis and facial paresis or other syndroms indicative of brain stem damage (dysarthria and speech impairment, vertigo, nausea, vomiting).

Inclusion criteria were pretreatment NECT immediately followed by MDCTA and available pretreatment hematocrit. Twenty-one patients were excluded because of prior contrast injection or poor image quality. The analyzed patient group consisted of 102 patients (39 women and 63 men) with a mean age of 70 +/- 14 years. Additional DSA was performed in 11 patients. This study was compliant with the Declaration of Helsinki and conducted with Ethics committee approval (Ethik-Kommission der Ärztekammer Hamburg WF-040/13). Due to the retrospective nature of this study and the poor clinical condition of the majority of the included patients the ethics committee waived informed written consent of the included patients. Patient records and information was anonymized and de-identified prior to analysis.

### Computed tomography

All patients were examined with the same CT scanner using 256-slice multidetector computer tomography (Brilliance iCT, Philips, Best, The Netherlands). For NECT the following scan parameters were used: collimation at sequential acquisition 16 x 0.625, tube voltage 120 kVp, tube current 333 / 310 mAs (infratentorial / supratentorial). Reconstructed slice thickness was 5 mm supratentorial and 2.5 mm infratentorial. MDCTA was performed after administration of 45 ml of nonionic contrast medium with an iodine concentration of 400 mg/ml (Imeron 400, Bracco Altana Pharma, Milan, Italy) through an 18-gauge peripheral intravenous catheter into a cubital vein at an injection rate of 4 ml/s. The following scan parameters were used: collimation 64 x 0.625, rotation time 0.4 s, tube current 300 mAs/slice, tube voltage 120 kVp. Scanning range was planned in a caudocranial direction. Bolus-tracking software was used to acquire images at peak contrast arrival.[8]

### Image Analysis

All NECTs were independently reviewed by two observers (M.E., M.G.) with more than four years of experience in neuroradiology that were employed at the same institution and were trained in advance. Both reviewers were asked to make a final judgment regarding the presence or absence of HBAS on NECT. Afterwards they performed HU measurements in the basilar artery by placing a circular region of interest (ROI) covering the whole basilar artery on a transverse slice. Care was taken to ensure that the boundaries of the ROI did not extend beyond the margins of the artery and that no calcified plaque was included.

The readers knew that all patients were clinically suspected of BAO, but were unaware of other clinical and imaging findings and had not participated in the treatment of any of the patients included in the study. Each reviewer was blind to the assessment of the other reader.

**Fig 1:**
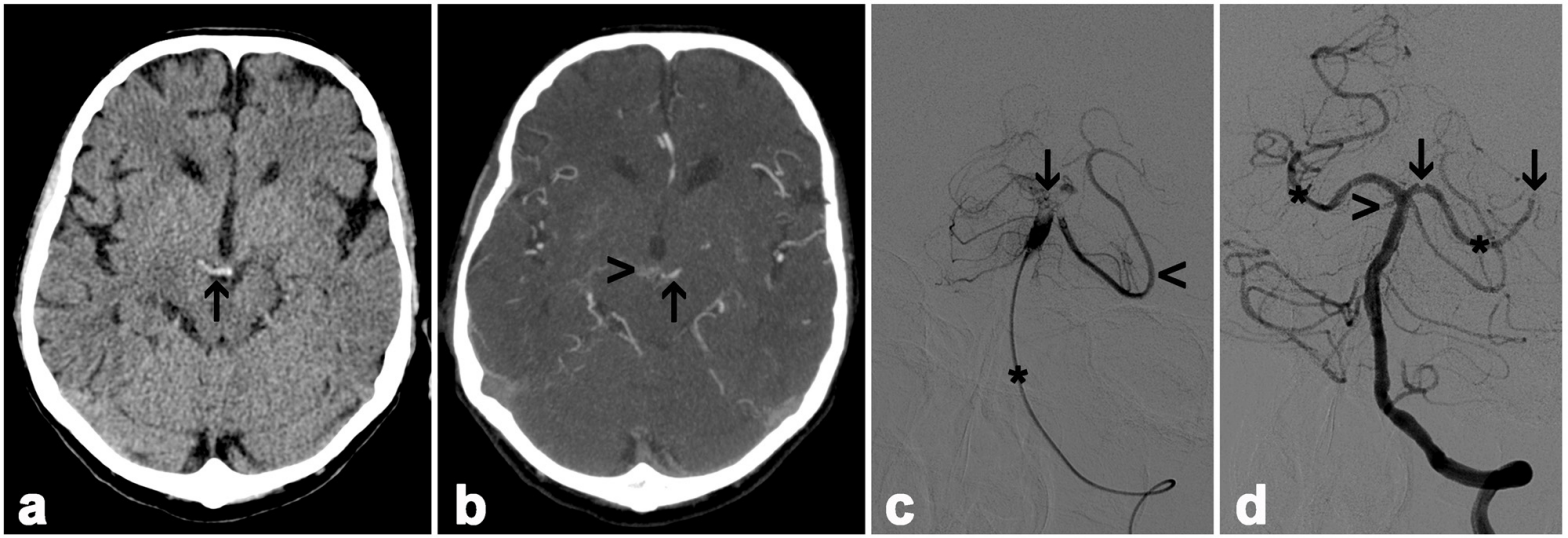
NECT (a), MDCTA (b), and DSA (c and d) of a 72 years old female. Transverse NECT demonstrates HBAS with extension into basilar branches (arrow in a). MDCTA confirmed BAO (b) and revealed multiple small collateral vessels (arrowhead) and preserved perfusion of the left superior cerebelli artery (arrow). DSA with the tip of the catheter placed in the middle basilar artery (asterisk) revealed occlusion of the basilar tip, both posterior cerebral arteries and the right sided superior cerebellar artery. The left sided superior cerebellar artery is still perfused (arrowhead in c). In this patient mechanical thrombectomy led to reperfusion of the basilar artery (d) and both posterior cerebral arteries (asterisk). Residual thrombus can be detected in the right posterior cerebral artery (arrow).

To evaluate intraobserver agreement one reviewer (M.E.) performed a second image analysis with a time interval of 4 weeks in an attempt to diminish recall bias. For exclusion of BAO both reviewers performed a consensus reading by analyzing MDCTA and DSA data sets (Fig 1).

For all patients included in this study the etiological diagnosis that was finally selected by the clinicians were derived from the medical reports.

### Statistical Analysis

To evaluate intra- and interobserver reliability of visual detection of HBAS on NECT Cohen’s kappa coefficient was calculated. A kappa value of up to 0.19 indicated positive but poor agreement, 0.21–0.40 fair agreement, 0.41–0.6 moderate agreement, 0.61–0.8 good agreement, and greater than 0.8 very good agreement [9].

To investigate inter- as well as intra-observer correlation and agreement for HU measurements, intra-class correlation coefficient (ICC)[10] and Bland-Altman analysis (BAA)[11] were applied. The following ICC interpretation scale was used: poor to fair (below 0.4), moderate (0.41–0.60), excellent (0.61–0.80), and almost perfect (0.81–1).[12]

DSA or, if not conducted, MDCTA served as reference standard for calculation of sensitivity, specificity, positive predictive value (PPV), and negative predictive value (NPV) for the detection of BAO by visual assessment, HU measurements, HU/Hct ratio and combination of visual assessment and attenuation measurements.

We used receiver operating characteristic (ROC) curve analysis to determine the optimal cut-off points to differentiate between patients with BAO and without BAO by HU measurements and HU/Hct ratio. The cut-off value derived from ROC curves at the point of highest accuracy was utilized to assess mean sensitivity, specificity, PPV, and NPV for the detection of BAO by HU measurements and HU/Hct ratio on NECT.

We conducted all statistical analyses by using commercially available software tools (MedCalc for Windows, Excel, Microsoft Corporation, Redmond WA USA and Mariakerke, Belgium).

## Results

### Descriptive statistics

Each final etiological diagnosis is shown in Table 1. BAO was detected on MDCTA in 16 patients. Median age of patients with BAO was 75 +/-12 years and without 70+/- 14 years. 43% of NECT/MDCTA examinations revealed cerebral pathology (BAO, 16%; posterior cerebral artery stroke, 6%; media cerebral artery stroke, 6%; basilar artery stenosis, 5%; cerebral embolism, 4%; vertebral artery stenosis, 2%; posterior reversible encephalopathy syndrome, 2%; intracranial hemorrhage, 2%). 11 patients underwent additional DSA, which confirmed BAO in 10 cases and showed a basilar stenosis in one case. The mean Hct in patients with proven BAO was 36.1% +/- 10.8% and without BAO 36.1 +/- 6.9%.

**Table 1:** Final etiological diagnosis.

Diagnosis	n	*%*
basilar artery occlusion	16	*16*
intoxication	12	*12*
unknown	11	*11*
seizure	11	*11*
posterior cerebral artery stroke	6	*6*
media cerebral artery stroke	6	*6*
myocardial infarction	5	*5*
transient ischemic attack	5	*5*
septic shock	5	*5*
basilar artery stenosis	5	*5*
cerebral embolism	4	*4*
dehydration	3	*3*
vertebral artery stenosis	2	*2*
posterior reversible encephalopathy syndrome	2	*2*
intracranial hemorrhage	2	*2*
hepatic encephalopathy	2	*2*
aspiration	2	*2*
aortic dissection	1	*1*
syncope	1	*1*
vestibular paralysis	1	*1*

### Intra- and interobserver reliability

**Fig 2:**
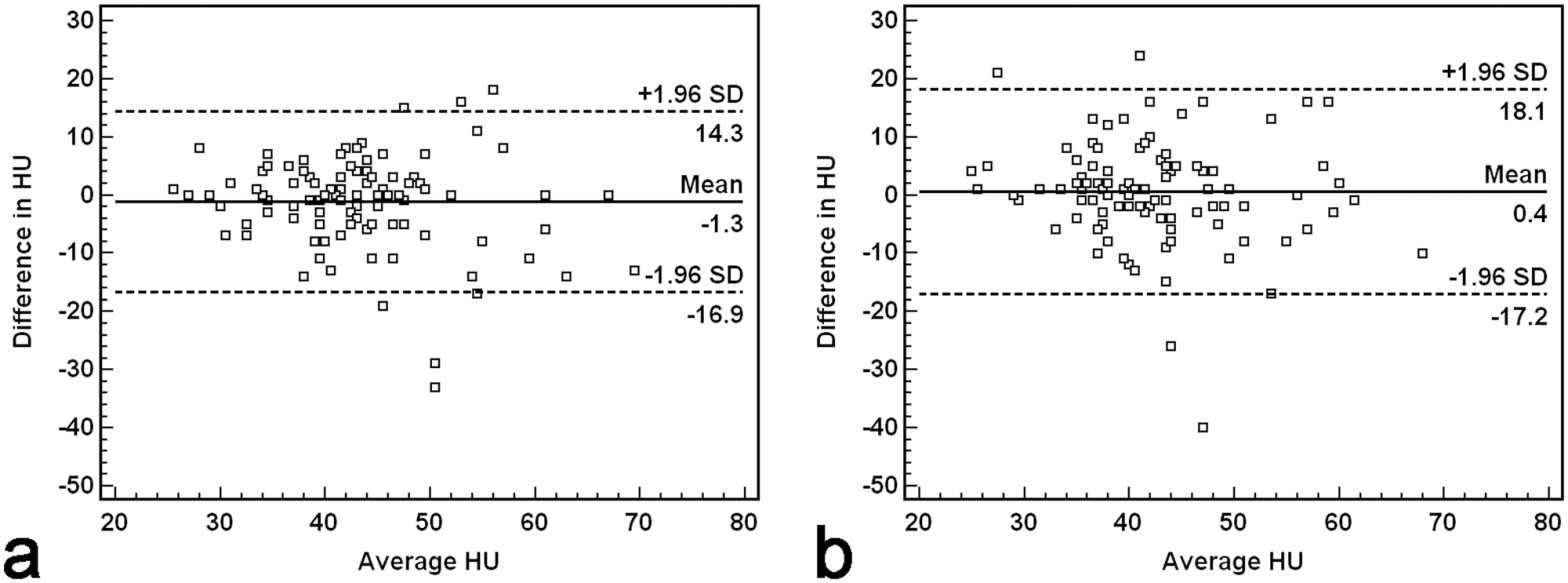
Bland-Altman plots of intra- (a) and interobserver agreement (b) for Hounsfield unit measurements performed in the basilar artery.

Good intraobserver reliability (Cohen´s kappa coefficient: 0.68) and moderate interobserver reliability (Cohen´s kappa coefficient: 0.60) were found for visual detection of BAO. Quantitative assessments with HU measurements showed excellent intra- (ICC: 0.62) and moderate interobserver correlation (ICC: 0.56). BAA revealed limits of agreement ranging between +/- 15.6 HU (intraobserver) and +/- 17.6 HU (interobserver) (Fig 2 and S1 Dataset).

### ROC and thresholds to identify BAO with density measurements and HU/Hct ratio

**Fig 3:**
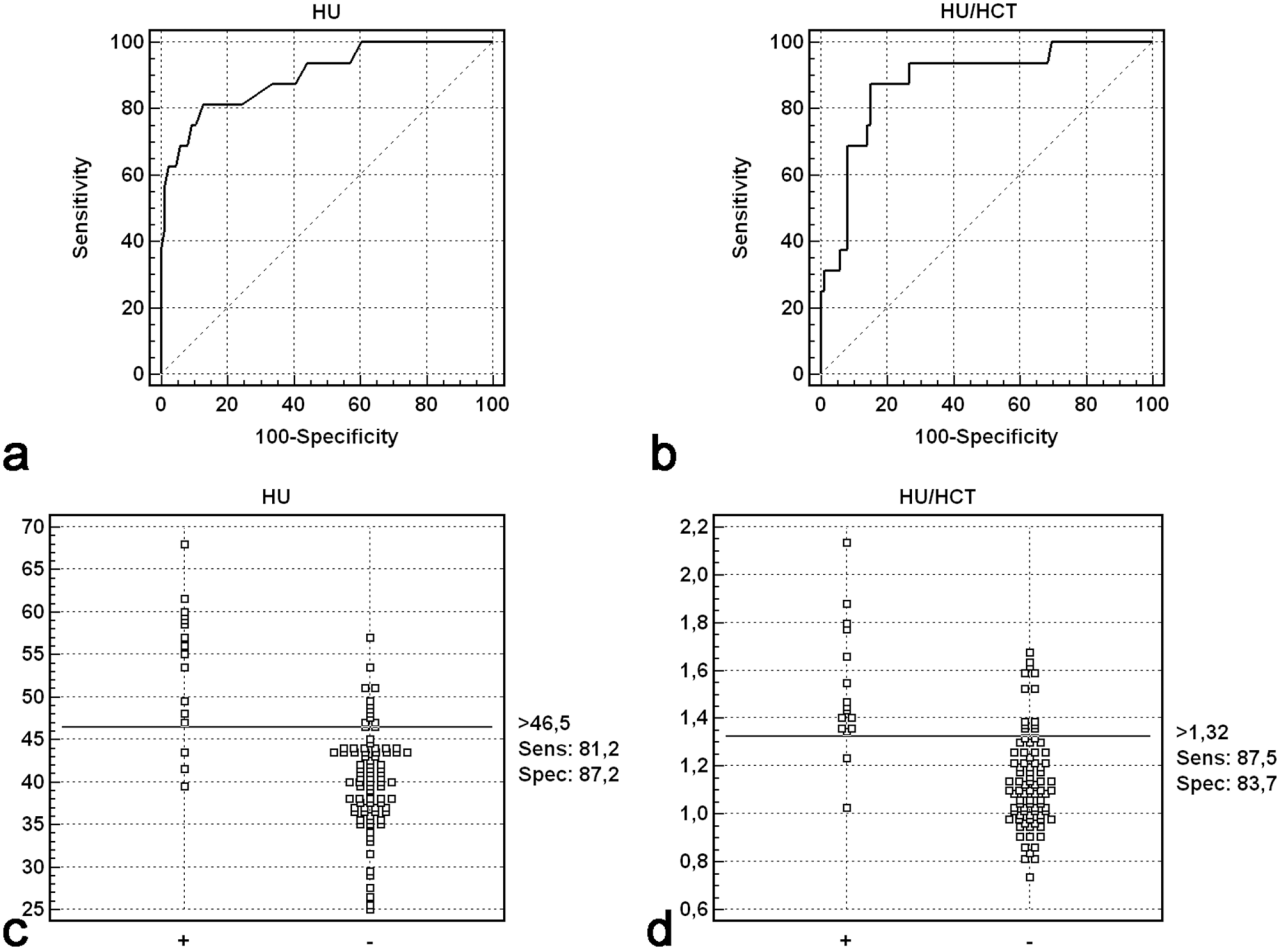
Receiver-operating characteristic (ROC) curve (a and b) and dot plot analysis (c and d). ROC analysis derived from calculated differences in Hounsfield unit (HU) measurements and hematocrit (Hct) corrected HU measurements of occluded (+) and non-occluded (-) basilar artery.

The highest accuracy for the detection of BAO was achieved for cut-off values of >46.5 HU for HU measurements and >1.32 HU/Hct for HU/Hct ratio (Fig 3 and S1 Dataset).

### Sensitivity, specificity, PPV, NPV and accuracy

Table 2 shows sensitivity, specificity, PPV, and NPV of visual assessment, attenuation measurements, Hct/HU ratio and combination of visual rating and attenuation measurements for the detection of HBAS. Accuracy was 89% for visual assessment, 88% for attenuation measurements, 84% for Hct/HU ratio, and 83% for combination of visual rating and attenuation measurements.

**Table 2:** Sensitivity, specificity, positive predictive value, and negative predictive value.

	Sensitivity	Specificity	PPV	NPV
**visual**	81%(54 to 95%)	91% (82 to 96%)	62% (39 to 81%)	96% (89 to 99%)
**> 46.5 HU**	81% (54 to 95%)	87% (78 to 93%)	54% (33 to 74%)	96% (88 to 99%)
**> 1.32 HU/Hct**	88% (60 to 98%)	84% (74 to 90%)	50% (31 to 69%)	97% (90 to 100%)
**visual or > 46.5 HU**	94% (68 to 100%)	81% (71 to 89%)	48% (31 to 67%)	99% (91 to 100%)

Sensitivity, specificity, positive predictive value (PPV), negative predictive value (NPV) with 95% confidence intervals (CI) of visual assessment, Hounsfield (HU) measurements, hematocrit (Hct) corrected HU measurements and combination of visual assessment and HU measurements for the detection of basilar artery occlusion.

## Discussion

In contrast to the previous studies, our study included the largest patient collective with clinically suspected BAO, who underwent NECT and additional DSA and/or MDCTA as reference standard. Moreover, sensitivity, specificity, PPV, NPV, and accuracy were calculated for four different approaches for the first time: Visual assessment alone, HU measurement alone, combination of visual assessment and HU measurement, and Hct/HU ratio. The key finding of our study is the increased sensitivity and NPV for the detection of BAO in NECT with a HU-threshold of 46.5 additionally to visual evaluation alone. However, despite the improved sensitivity, we would like to emphasize that the HBAS on NECT by itself is not accurate enough to be used as the only decision-making tool in BAO and cannot replace angiography but should rather be used to recognize the urgent need for further vessel imaging. Thus our results are probably most helpful in regions where additional investigations are not readily available and a transfer to a facility offering complementary imaging studies should be initiated.

This analysis was done in a typical consecutive patient population with clinically suspected BAO. The final etiological diagnosis of BAO was established in 16/102 (16%) of all cases. In a previous study determining the utility of HBAS in a similar patient group presenting with symptoms of posterior circulation stroke, BAO was proven by CTA in 15% of the 95 patients, however they unfortunately do not present the etiology of the remaining 81 patients.[6] In our study group, the 2^nd^ most common diagnosis was intoxication (Table 1). Concerning visual assessment of the HBAS on NECT our findings are similar to previous studies reporting a low sensitivity ranging from 57–71%[5,6,13] and a high specificity (71%-98%).[5,6,13] In screening for BAO, a high sensitivity is more important than a high specificity as missing the diagnosis would have dramatic consequences.

In our study the highest sensitivity was achieved by visual assessment and additional quantitative attenuation measurements applying a density threshold of 46.5 HU. This is comparable to previous studies analyzing the sensitivity of the dense media sign as an indicator for occlusion of the middle cerebral artery achieving the highest sensitivity by combining visual assessment and additional quantitative density measurements.[14]

The optimal cut-off point of 46.5 HU in our study is close to previous studies reporting that attenuation values above 45 HU are suggestive of BAO.[5,7]

Connell et al. observed that especially less experienced observers achieved a pronounced improvement of the diagnostic accuracy after measuring BA attenuation.[5] In another study the optimal quantitative threshold for predicting BAO was a basilar artery density greater than 55 HU.[13] However, this study analyzed just seven cases with BAO. The optimal threshold might vary for different CT scanners and scan parameters, as HU values depend on filtration and kilovolt peak.[15] Therefore our results must be interpreted with caution and our cut-off value of 46.5 HU might not be applicable on every CT-scanner. In contrast to previous studies [5,6] using different scanners with varying parameters, all patients included in our study were examined on the same CT scanner with the same scan parameters to eliminate variations in the HU values. As another strength of our study all patients had a NECT followed immediately by MDCTA and in 11 cases an additional DSA was performed. Previous studies reported an excellent correlation between CTA and DSA for identifying occlusion or stenosis of the large intracranial arteries.[16], [17] However the proportion of patients with BAO undergoing both imaging modalities in these studies is small with only six [16] respectively seven patients [17]. Despite lacking evidence we assume MDCTA to be highly accurate in detecting BAO and the results of our study underline this assumption, as DSA confirmed the MDCTA findings in all 11 patients who had undergone both examinations.

A major strength of our study is the availability of vessel imaging in all patients with clinically suspected BAO. This is in contrast to previous studies that might suffer from partial verification bias since the reference examination was not applied consistently to confirm negative results of the initial CT scan. Thus in the study of Connell et al. only the group with proven BAO was verified by CTA.[5] In the control group BAO was excluded if there was absence of BAO on the follow-up scan. Their approach additionally bears the risk of incorporation bias as the index test under evaluation is also part of the reference test used to establish the final diagnosis. With regard to reliability, visual assessment as well as attenuation measurement proved to be reliable approaches in the detection of BAO in our study. However, inter- and intraobserver reliability was lower in our study compared to studies analyzing the hyperdense media sign.[14] This might be due to the fact that the identification of the HBAS is more challenging as the basilar artery is imaged in cross-section and there is no paired vessel to compare it with.

For NECT we constantly applied a slice thickness of 5 mm as it was the standard in most institutions in the period of examination. Interestingly, a recent systematic review and meta-analysis observed that CT slice-thickness was significantly associated with sensitivity but not specificity of hyperdense artery sign and was inversely proportional to the year of article publication.[18] Thus, sensitivity of the HBAS might be improved with thin-slice volumetric NECT.

To the best of our knowledge, our study is the first one evaluating the value of HU/Hct ratio for the detection of BAO on NECT. Similar to previous studies analyzing the usefulness of attenuation measurement and HU/Hct ratio in diagnosing middle cerebral artery occlusion [14] or acute cerebral venous sinus thrombosis [19] we found only a minor difference between the accuracy/sensitivity of the HU/Hct ratio and the accuracy/sensitivity of the HU measurement alone. This might be due to the fact that our study population suffering from diseases such as dehydration, septic shock or intoxication tended to have similar Hct levels to patients with BAO.

To create a realistic clinical scenario, only patients with clinically suspected BAO were included. This elevated pretest probability might explain the higher accuracy in our study and in the study of Goldmakher et al.[6] compared to a previous study [5] examining a clinically heterogeneous group of patients with CTA-proven BAO and a control group without any cerebral vessel occlusion. However, we chose this study design to create a real-life clinical scenario, as physicians interpreting the CT scan should be, and usually are, aware of the clinical presentation.

In our study, the combination of visual assessment and additional attenuation measurement improves the diagnostic sensitivity in the detection of BAO on NECT. However, given a specificity of 81%, absence of HBAS does not predict normal arterial patency and additional angiography should be considered. Moreover, it is important to differentiate between the detection of an acute basilar artery occlusion and the clinical diagnosis of brain stem ischemia. Ischemia of the brain stem can also occur if there is no proof of basilar artery occlusion on imaging, for example when recanalization already occurred or in the case of small artery occlusion. Thus the HBAS cannot be used to rule out brain stem ischemia.

The greatest utility of our results is in the situation in which a patient with clinically suspected acute BAO presents to a medical center where angiographic imaging is not currently available. In such circumstances, our approach can enable physicians performing NECT to make the best use of all available imaging information. Visual assessment of HBAS confirmed by additional HU measurements provides substantial confidence that there is a high likelihood of the diagnosis of BAO and urgent need for additional angiography such as CTA, MRA or transfer to a facility offering complementary imaging studies and treatment resources.

## Conclusion

In patients with clinically suspected acute BAO the combination of visual assessment and additional attenuation measurement with a cut-off value of 46.5 HU is a reliable approach that improves the sensitivity in the detection of BAO on NECT and guide radiologist in choosing further imaging to confirm vessel occlusion.

## Supporting Information

S1 Dataset Patient characteristics and density measurements. (XLSX)
